# Patient dose measurement in common medical X‐ray examinations in Iran

**DOI:** 10.1120/jacmp.v17i1.5860

**Published:** 2016-01-08

**Authors:** Behrouz Rasuli, Ali Mahmoud‐Pashazadeh, Mohammad Ghorbani, Raheleh Tabari Juybari, Mozafar Naserpour

**Affiliations:** ^1^ Department of Radiology Technology Behbahan Faculty of Medical Sciences Behbahan Iran; ^2^ The Persian Gulf Nuclear Medicine Research Centre, Bushehr University of Medical Sciences Bushehr Iran

**Keywords:** patient dose, dose reference level, ESAK, X‐ray examination, conventional radiology

## Abstract

The main purpose of this study was to investigate patient dose in the chest (PA/AP/LAT) and skull (PA/AP/LAT) X‐ray examinations, as frequent procedures. The study was performed in eight public hospitals of Khuzestan province, Iran. Patient dosimetry was conducted on 567 standard patient X‐ray examinations (males: 61.2%, female: 38.2%). Dosimetry protocol in this study was indirect method, according to the International Atomic Energy Agency (IAEA) Technical Reports series No. 457. Patients weighing 70±10 kg were considered as standard. In the indirect dosimetry approach, exposure parameters such as kVp, mAs, focal film distance (FFD), and tube outputs recorded during data acquisition were used for calculating incident air kerma on the patient's skin, entrance surface air kerma (ESAK) that is recommended by the IAEA as the most appropriate patient dosimetry quantity in simple radiographic examinations. This survey reveals significant variations in the radiological practice. Results showed that the parameters set by radiologic technologists change in a wide range: mAs varied from 2 to 80 for skull PA, 2 to 202 for chest LAT, and FFD varied from 50 to 180 for skull LAT projection. The study showed that patient doses in three chest projections exceed the IAEA and European Commission dose reference levels (EC DRLs) — 1.0, 1.12, and 2.20 mGy for chest PA, chest AP, and chest LAT, respectively. Results also showed that mean ESAKs of patients in skull projections were generally lower than the IAEA and EC DRLs, 1.5, 1.72, and 2.25 for skull LAT, skull AP, and skull PA, respectively. This study provides evidence that dose reduction in the simple X‐ray examinations is feasible by updating clinical audits and implementation of systematic quality assurance (QA) and quality control (QC) programs. The authors recommend that DRLs obtained in this study can be used as local DRLs in Khuzestan area and dose surveys must be performed in all provinces to establish national dose reference levels (NDRLs) in Iran.

PACS numbers: 87.53.Bn, 87.57.uq, 87.59.B

## I. INTRODUCTION

Annually, a considerable number of medical diagnostic procedures are being performed using X‐ray systems worldwide. In Iran, for example, 18,867,000 X‐ray examinations were carried out on 12,963,000 patients in 2003, representative of an average of almost 1.5 examinations per patient. Based on Iran's population in 2003, that is equivalent to 363 examinations per 1,000 inhabitants.^(1)^ Widespread fast‐growing demand for X‐ray examinations by physicians in the developing countries, as in Iran, and increasing the number of X‐ray machines in the past 10 years have led to increase the number of X‐ray examinations per unit of population. Therefore, it is of pivotal importance to implement radiation protection programs based on three main principles of justification, optimization, and dose constraint in radiographic practices to take advantage of diagnostic X‐ray while effectively mitigating its associated risks.

International Commission on Radiation Protection (ICRP) declared that the diagnostic reference levels (DRLs) are already being used in medical diagnosis to indicate whether the levels of patient dose from a specified imaging procedure are unusually high or low in comparison to the predefined criteria. If so, a local review should be initiated to determine appropriate protective action. This means that cooperation between national authorities and professional bodies is necessary to establish national diagnostic reference levels (NDRLs), taking into account the prevailing economic and societal circumstances, as well.^(2)^ Regular control and dosimetry can help the physician and physicist to ensure that the dose received by patients who undergo radiologic procedures is in accordance with the ALARA (As Low As Reasonably Achievable) principle and does not exceed the amount required to obtain favorable radiographic scan.

Entrance Skin Air Kerma (ESAK) is recommended by the IAEA as the most appropriate patient dosimetry quantity in simple radiographic examinations, primarily due to the convenience of measurement, easy comparison with other studies in different countries or DRLs, and proportionality to patient effective dose that is used to find the probability of radiation‐induced complications.^(3)^

There is neither an established national diagnostic reference level nor a systematic recording of patient radiographic information in Iran. Thus a comprehensive national plan should be established to determine NDRLs. Iran also was not involved in the international project of patient dose survey, under the supervision of the IAEA, in the year 2004, which makes these types of studies more important.^(3)^ While during the past decades several patient dose survey have been performed around the world,^4^, ^5^, ^6^, ^7^, ^8^, ^9^, ^10^, ^11^ but a few noteworthy studies have been conducted on patient dose in Iran.^1^, ^12^, ^13^, ^14^, ^15^ The authors believe that patient dose survey must be performed in all provinces, as Local Dose Reference Levels (LDRLs) to complete the National Dose Reference Levels (NDRLs) puzzle. The first result of a patient dose survey in Khuzestan province of Iran, as undeveloped area, is given in this study. This work is in progress and further analysis will be reported subsequently.

Therefore the main purpose of this study was to investigate patient dose in chest (PA/AP/LAT) and skull (PA/AP/LAT) radiographic examinations, frequent procedures that are taken in eight public hospitals of Khuzestan province, in order to evaluate how the ICRP principle of optimization could be implemented in practice, and to compare our situation with other provinces and countries.

## II. MATERIALS AND METHODS

In this study, performed January to May 2014, ESAKs value of patients referred to eight public hospitals located in seven selected cities in Khuzestan province of Iran were evaluated. These hospitals were selected according to high‐patient‐load of the X‐ray examinations, as well as considering uniform geographic distribution of these centers in the province area. Due to ethical consideration the hospital names were changed to H1–H8. This work is limited to the study of six most common X‐ray procedures, chest (AP/PA/LAT), and skull (AP/PA/LAT), in eight conventional radiology systems. These procedures were selected based on their frequencies and contribution to the collective dose delivered to the public. Dosimetry protocol in this study was indirect assessment, according to the IAEA Technical Reports Series No. 457.^(16)^

### A.1 Pre‐experimental phase

Quality control tests were performed on X‐ray machines as necessary pre‐experimental phase. Ten standard QC tests, including voltage accuracy and reproducibility, exposure time accuracy and reproducibility, linearity of the tube output (time and milliampere), filtration (HVL), tube output (70 kV at FSD=100 cm). Reproducibility of the tube output and beam alignment were calculated and assessed based on the Institute of Physics and Engineering in Medicine protocol, IPEM Report No. 91.^(17)^ This survey was conducted using a state‐of‐the‐art calibrated Barracuda X‐ray multi‐purpose detector (MPD) (RTI Electronics AB, Mölndal, Sweden) for dosimetry tests, Alpha test phantom (PEHA med. Geräte GmbH, Sulzbach, Germany) for beam alignment test, pure aluminum HVL filter (RTI), dosimeter and HVL measuring stand, typical digital scale for measuring patients' weight, and flexible measuring tape to measure distances and patient thickness.

### A.2 Data collection method

**Figure 1:**
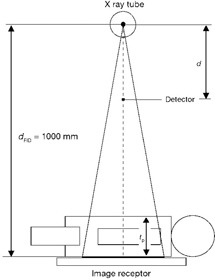
Geometry used for the calculation of incident air kerma and entrance skin air kerma.

Patients were weighed immediately before the radiography to ensure compliance with the standard patient, 70±10 kg. Patient thickness and exposure parameters were recorded. Based on measurement method illustrated in Fig. 1, patient thickness ($t_{p}$) was measured with measuring tape at the center of X‐ray beam. Measurement of $t_{p}$ was performed from table top to skin surface of the organ under examination, taking into account the field orientation (AP,PA or LAT).

The comprehensive information, including institutional data (hospital name, room number and annual patient load), X‐ray machine data (kVp, HVL, FSD, output in some clinical kVps, generator type, grid usage, image receptor type, film size, production year, screen‐film speed class and exposure setting), and patient information (gender, weight, height, age, organ thickness, examination type and projection) were recorded in the self‐designed forms. All the data gained through direct observation by previously trained radiologic technologists of the center.

Based on the IAEA method, at least 10 standard patients, including both males and females, should be assessed for any procedure. Patients weighing 70±10 kg were considered as standard, and obese patients (BMI≥;30) and infants were excluded from this survey. Since dosimetry was performed on six examinations in eight radiology departments and a minimum of 10 patients were evaluated in each examination, at least 480 patients were assessed. The actual number of examinations was 567 cases (some patients had more than one radiograph and sample size in some procedures was more than 10 patients).

### B. Indirect dosimetry approach

In the indirect dosimetry approach that is shown in Fig. 1, exposure parameters recorded during data acquisition, such as kVp, mAs, and distances, are used for calculating incident air kerma. In this method, tube output (Y(d)) is measured in predetermined distance from the tube focal spot by placing the detector at the reference point. The tube output is affected by kVp, mAs, FFD, HVL, and d (distance between the detector and the tube focal spot). X‐ray tube output can be calculated using Eq. (1):

(1) Y(d)=K(d)/mAs

where Y(d) and k(d) are, respectively, the X‐ray tube output and air kerma at distance *d* from the tube, and *mAs* is the tube current‐exposure time product.

Incident air kerma from the exposure can be calculated directly using the tube output and inverse square law using Eq. (2):

(2) $$K_{i}=Y(d)\times mAs{\times (d/(d_{FTD}-t_{p}))}^{2}$$

where $K_{i}$ is the incident air kerma, *d* is the distance between the detector and the tube focal spot, $d_{\text{FTD}}$ is distance between the tube focal spot and tabletop, and $t_{p}$ shows thickness of organ under examination that was measured for each patient separately.

Entrance skin air kerma can be calculated by the product of the calculated values of the incident air kerma and backscatter coefficient (B), using Eq. (3):

(3) $$ESAK=K_{i}\times B$$

A backscatter factor is defined as the quotient between the absorbed dose on the surface of the patient (skin) to the absorbed dose at the same point in space in the absence of the patient. This parameter gives the factor by which the radiation dose at a determined point in the air is increased by radiation scattered to the same point from the patient. Appropriate backscatter factor based on the measured HVL (in the pre‐experimental QC phase), kVp, and the field size can be found in the IAEA Technical Reports Series No. 457, Appendix VIII.^(16)^

In order to perform statistical analysis, all measurements, including dosimetry in reference point, measurement of output in the clinical range of kVps, calculation of incident air kerma and entrance skin air kerma were repeated at least three times to reduce the likelihood of errors or anomalous results. Then mean value, percent of error, variation coefficient, standard deviation, and min‐max values were calculated using SPSS v16. The results of this study were compared with similar data reported by other researchers, and the IAEA and EC DRLs.^18^, ^19^

## III. RESULTS

Technical characteristics of imaging systems and center's information in which patients were studied are presented in Table 1. Information on these devices was obtained by direct checking of the equipment. As it can be seen from Table 1, all radiographic equipment was more than 10 years old, except the X‐ray machine located in the H3 hospital. Manual exposure setting is so common, automatic exposure control (AEC) systems either did not exist or could not be used. Most of the centers were using 400‐speed screen‐film (SF) cassettes.

Prior to starting the study, quality control tests according to the IPEM Report 91 criteria were performed on all systems. Quality control criteria and results, shown in Table 2, indicate that all equipment successfully passed nearly all acceptance standards. Tube output in terms of routine clinical kilovoltages in all centers is shown in Table 3. It is noteworthy that, instead of using the extrapolation method, tube outputs were measured by placing an MPD dosimeter on a scatter‐free support on the table at FSD=100 cm. 4, 7 provide descriptive statistics of the patient's weight, exposure parameters, and examination frequency in the various hospitals. These data were collected by the radiologic technologists of each center, then precisely entered in the predetermined forms.

**Table 1:** Imaging department's information and X‐ray devices data.

*Hospital Code*	*Manufacturer*	*Production Year*	${\text{kVp}}_{\text{max}}$	*Exposure Setting*	*Generator Type*	*Film Type*	*Annual Patient Load*	*Output at 80 kVp*	*HVL (mm) at 70 kVp*	*Image Receptor*
H1	Shimadzu	1994	150	Manual	1‐phase	AGFA	27000	41.8	2.2	SF‐400
H2	Varian	1997	150	Manual	3ph‐12pu	CEA	57600	72.7	2.8	SF‐400
H3	Varian	2011	150	Manual	3ph‐12pu	Fujifilm, KODAK	36000	104.3	1.9	SF‐400
H4	Varian	2000	150	Manual	3ph‐12pu	AGFA	13200	58.8	3.5	SF‐400
H5	Shimadzu	1999	150	Manual	3ph‐12pu	AGFA, CEA, Fujifilm	36000	–	2.9	SF‐400
H6	Villa Medical Systems	1990	150	Manual	1‐phase	Retina, Fujifilm, CEA	36000	20.2	2.7	SF‐400
H7	Varian	2003	150	Manual	3ph‐12pu	AGFA	36000	–	3	SF‐400
H8	Toshiba	1999	150	AEC	1‐Phase	Kodak	54000	35	3.2	SF‐400

**Table 2:** Hospitals H1–H8 radiology units meeting the IPEM criteria.

	*Good*		*Normal*		*Poor*	
*Test*	*Criteria*	*Result*	*Criteria*	*Result*	*Criteria*	*Result*
Voltage accuracy	<±5%	All except H6	±5% to ±10%	H6	>±10%	
Voltage reproducibility	<±5%	All	±5% to ±10%		>±10%	
Exposure time accuracy	<±5%	All except H4	±5% to ±10%	H4	>±10%	
Time reproducibility	<±5%	All	±5% to ±10%		>±10%	
Linearity of the tube output	<±5%	All except H1	±5% to ±10%	H1	>±10%	
Filtration (HVL)	>2.3 mm AL	All except H1, H3			<2.3 mm AL	H1, H3
Tube output (80 kVp)	43‐52μGy/mAs	All except H3, H6	26‐43μGy/mAs		<26μGy/mAs	H3, H6
			52‐69μGy/mAs		>69μGy/mAs	
Output Reproducibility	<±5%	All	±5% to ±10%		>±10%	
Beam alignment	<±1% from FFD	H1, H3, H5, H8	±1% to ±2%	H2, H4, H6, H7	>±2% from FFD	

**Table 3:** Tube outputs in terms of routine clinical kVp in all hospitals (mAs=10).

*kVp*	*H1*	*H2*	*H3*	*H4*	*H5*	*H6*	*H7*	*H8*
55	16.3	32.4	56.5	25	28.2	9.4	23.4	13
60	22.6	39.6	64.6	31.2	34.6	11.9	28.3	16.8
65	27.3	47.2	74.9	36.9	41	13.9	33.5	–
70	31.8	55.3	85.1	44.3	47.9	16.4	38.9	22.8
75	36.5	–	94.8	51.2	55	17.8	44.5	28.8
80	41.8	72.7	104.3	58.8	–	20.2	–	35
85	–	–	–	–	–	29.9	–	42.4

In the case of skull LAT procedure, the number of patients was less than standard (total of 29 cases), but in the chest PA procedure there were more (total of 178 cases). The most‐ and least‐frequent exams were chest PA and skull LAT, respectively. In comparison to other X‐ray centers, the H3 center admitted more patients (89 cases) than others during the study. The least number of patients was referred to the H6 center (52 cases). Detailed information about the number of examinations in any hospital is presented in Table 8. Dose assessment was conducted on a total of 567 examinations (males: 61.2%, females: 38.2%). Exposure parameters and the number of patients in each exam are given in Table 9.

The mean ± SD value, minimum‐maximum range, and maximum‐to‐minimum ratio of ESAKs obtained in three different projections of the chest (AP/ PA/LAT) and skull (AP/ PA/LAT) examinations for any hospitals separately are shown in 10, 11, respectively. Table 11 summarizes ESAK statistical parameters in all imaging centers of this study. As a comparison, Table 12 also shows some similar studies and dose reference levels set by the EC and IAEA. Care must be taken that EC dose reference levels are presented as a 3rd quartile.

**Table 4:** Patients and examinations characteristics in hospitals H1 and H2, (mean value and min‐max range).

	*H1*					*H2*				
	*Patient Data*		*Exposure Parameters*			*Patient Data*		*Exposure Parameters*		
*Exam*	*No*.	*Weight*	*kVp*	*mAs*	*FFD (cm)*	*No*.	*Weight*	*kVp*	*mAs*	*FFD (cm)*
Skull AP	5	68±16	70 (70−71)	63 (30−75)	119 (89 150)	10	69±13	65 (60−68)	17 (14 22)	100 (100 100)
Skull PA	6	50±25	70 (69−70)	68 (30−75)	130 (120 150)	7	71±7	66 (65−73)	20 (14−25)	102 (100 115)
Skull LAT	–	–	–	–	–	–	–	–	–	–
Chest AP	11	61±10	64 (60−66)	20 (12−44)	101 (80−120)	7	76±15	63 (58−70)	20 (5−50)	109 (100 115)
Chest PA	28	67±12	68 (58−75)	34 (24−60)	115 (100−120)	34	70±14	67 (55−90)	19 (11 71)	111 (100 120)
Chest LAT	9	50±14	80 (74−82)	59 (36−75)	133 (120−150)	7	78±13	70 (68−73)	24 (16 32)	108 (100 120)
	Total Number of patients (male, female): 59 (30,29)					Total Number of patients (male, female): 65 (37,28)				

**Table 5:** Patients and examinations characteristics in hospitals H3 and H4, (mean value and min‐max range).

	*H3*					*H4*				
	*Patient Data*		*Exposure Parameters*			*Patient Data*		*Exposure Parameters*		
*Exam*	*No*.	*Weight*	*kVp*	*mAs*	*FFD (cm)*	*No*.	*Weight*	*kVp*	*mAs*	*FFD (cm)*
Skull AP	18	75±17	67 (55−75)	8 (2−20)	97 (57−110)	16	64±22	65 (60−67)	17 (8−20)	97 (70−110)
Skull PA	10	67±12	66 (62−70)	8 (2−16)	100 (90−110)	15	62±21	65 (60−74)	17 (8−26)	98 (90−110)
Skull LAT	7	63±12	64 (58−68)	10 (3−20)	89 (50−100)	7	58±28	61 (58−65)	12 (8−16)	98 (70−110)
Chest AP	19	71±15	66 (56−83)	7 (2−20)	97 (70−120)	17	73±15	65 (59−69)	14 (8−42)	126 (100−180)
Chest PA	18	67±17	68 (60−84)	12 (8−31)	114 (92−160)	16	73±7	67 (60−72)	13 (10−16)	146 (100−180)
Chest LAT	17	72±12	71 (50−82)	12 (3−25)	111 (100−180)	14	69±17	74 (67−78)	23 (13−51)	139 (115−160)
	Total Number of patients (male, female): 89 (63,26)					Total Number of patients (male, female): 85 (44,41)				

**Table 6:** Patients and examinations characteristics in hospitals H5 and H6, (mean value and min‐max range).

	*H5*					*H6*				
	*Patient Data*		*Exposure Parameters*			*Patient Data*		*Exposure Parameters*		
*Exam*	*No*.	*Weight*	*kVp*	*mAs*	*FFD (cm)*	*No*.	*Weight*	*kVp*	*mAs*	*FFD (cm)*
Skull AP	17	69±18	54 (50−58)	23 (10−40)	77 (70−98)	9	70±18	67 (65−66)	11 (10−12)	98 (95−100)
Skull PA	10	70±18	55 (50−58)	20 (5−40)	77 (70−120)	12	65±18	67 (64−74)	36 (24−40)	80 (75−85)
Skull LAT	–	–	–	–	–	5	73±8	65 (64−67)	35 (24−40)	78 (75−84)
Chest AP	21	78±13	57 (53−65)	38 (20−80)	133 (90−180)	6	55±6	66 (60−71)	16 (12−18)	84 (84−84)
Chest PA	21	64±12	57 (52−66)	37 (25−50)	180 (180−180)	20	66±11	71 (44−81)	19 (8−24)	173 (148−200)
Chest LAT	10	69±14	63 (50−72)	48 (20−80)	122 (75 180)	–	–	–	–	–
	Total Number of patients (male, female): 79 (43,36)					Total Number of patients (male, female): 52 (30,22)				

**Table 7:** Patients and examinations characteristics in hospitals H7 and H8, (mean value and min‐max range).

	*H7*					*H8*				
	*Patient Data*		*Exposure Parameters*			*Patient Data*		*Exposure Parameters*		
*Exam*	*No*.	*Weight*	*kVp*	*mAs*	*FFD (cm)*	*No*.	*Weight*	*kVp*	*mAs*	*FFD (cm)*
Skull AP	10	69±11	60 (55−65)	26 (6−32)	101 (90−126)	*1*	71±10	68 (65−72)	56 (45−70)	100 (100−100)
Skull PA	10	70±10	57 (53−60)	29 (6−32)	101 (90−126)	18	68±13	64 (58−70)	61 (32−80)	104 (50−180)
Skull LAT	–	–	–	–	–	10	66±12	60 (55−68)	50 (40−80)	108 (100−180)
Chest AP	11	76±10	66 (58−70)	15 (2−20)	124 (100−170)	11	74±19	73 (66−82)	30 (13−64)	180 (180−180)
Chest PA	21	68±8	67 (58−74)	17 (12−20)	112 (100−150)	20	76±18	72 (66−82)	37 (10−64)	180 (180−180)
Chest LAT	10	79±6	69 (64−75)	4 (2−16)	128 (100−152)	10	63±10	73 (68−78)	78 (202−64)	180 (180−180)
	Total Number of patients (male, female): 62 (48,14)					Total Number of patients (male, female): 76 (35,41)				

**Table 8:** Patient and exam characteristics in eight hospitals.

*Hospital Code*	*Skull AP*	*Skull PA*	*Skull LAT*	*Chest AP*	*Chest PA*	*Chest LAT*	*Total Number of Patients (male, female)*
H1	5	6	–	11	28	9	59 (30,29)
H2	10	7	–	7	34	7	65 (37,28)
H3	18	10	7	19	18	17	89 (63,26)
H4	16	15	7	17	16	14	85 (44,41)
H5	17	10	–	21	21	10	79 (43,36)
H6	9	12	5	6	20	–	52 (30,22)
H7	10	10	–	11	21	10	62 (48,14)
H8	7	18	10	11	20	10	76 (35,41)
TOT	92	88	29	103	178	77	567 (347,220)

**Table 9:** Exposure parameters based on exams (mean value and min‐max range) in all hospitals.

				*Grid Usage*		*Total Number of patients (male, female)*
*Examination*	*kVp*	*mAs*	*FFD*	*Yes*	*No*	
Skull AP	63 (50−75)	20 (2−75)	95 (57−150)	83%	17%	92 (55,37)
Skull PA	64 (50−74)	33 (2−80)	100 (50−180)	93%	7%	88 (60,28)
Skull LAT	62 (55−70)	28 (3−80)	98 (50−180)	100%	0%	29 (20,9)
Chest AP	64 (44−83)	20 (2−80)	125 (70−180)	75%	25%	103 (62,41)
Chest PA	67 (44−90)	24 (8−71)	140 (100−200)	100%	0%	178 (96,82)
Chest LAT	71 (50−85)	31 (2−202)	132 (75−180)	94%	6%	77 (54,23)

**Table 10:** ESAK for chest projections (AP/PA/LAT) in eight hospitals.

	*Chest AP Examination ESAK (mGy)*		*Chest LAT Examination ESAK (mGy)*		*Chest PA Examination ESAK (mGy)*	
*Hospital Code*	*mean (SD)*	*(min‐max) and max to min ratio*	*mean (SD)*	*(min‐max) and max to min ratio*	*mean (SD)*	*(min‐max) and max to min ratio*
H1	1.14 (1.28)	(0.45− 4.02), 8.9	3.16 (0.94)	(2.12− 4.34), 2.1	1.43 (0.54)	(0.71− 3.73), 5.3
H2	1.68 (1.06)	(0.29− 3.04), 10.5	3.47 (0.85)	(2.19− 4.58), 2.1	1.57 (1.12)	(0.61− 5.06), 8.3
H3	1.49 (1.83)	(0.26− 7.65), 29.4	2.04 (1.15)	(0.42− 5.19), 12.4	1.68 (1.05)	(0.44− 4.14), 9.4
H4	0.73 (0.56)	(0.36− 2.71), 7.52	1.42 (0.75)	(0.50− 3.13), 6.3	0.50 (0.29)	(0.26− 1.20), 4.6
H5	1.49 (0.84)	(0.45− 2.99), 6.6	4.80 (4.49)	(0.50− 13.74), 27.48	0.59 (0.19)	(0.32− 1.00), 3.1
H6	0.58 (0.12)	(0.50− 0.69), 1.4	–	–	0.17 (0.63)	(0.02− 0.27), 13.5
H7	0.63 (0.42)	(0.08− 1.43), 17.9	0.24 (0.24)	(0.04− 0.86), 21.5	0.87 (0.23)	(0.47− 1.35), 2.9
H8	0.49 (0.40)	(0.13− 1.44), 11.1	1.38 (0.90)	(0.83− 3.89), 4.7	0.55 (0.38)	(0.10− 1.44), 14.4

**Table 11:** ESAK for skull projections (AP/PA/LAT) in eight hospitals.

	*Skull AP Examination ESAK (mGy)*		*Skull LAT Examination ESAK (mGy)*		*Skull PA Examination ESAK (mGy)*	
*Hospital Code*	*mean (SD)*	*(min‐max) and max to min ratio*	*mean (SD)*	*(min‐max) and max to min ratio*	*mean (SD)*	*(min‐max) and max to min ratio*
H1	2.6 (0.70)	(1.46− 3.11), 2.1	–	–	2.30 (0.79)	(1.14− 3.07), 2.7
H2	1.76 (4.14)	(1.11− 2.38), 2.1	–	–	1.92 (0.60)	(1.27− 2.76), 2.2
H3	1.35 (1.06)	(0.28− 4.46), 15.9	1.93 (0.92)	(1.19− 3.63), 3.1	1.20 (0.76)	(0.24− 2.38), 9.9
H4	1.55 (8.19)	(0.39− 3.96), 10.1	0.99 (0.70)	(0.38− 2.49), 6.6	1.61 (0.79)	(0.39− 3.78), 9.7
H5	2.41 (1.05)	(0.61− 3.97), 6.5	–	–	2.67 (1.16)	(0.18− 3.75), 20.8
H6	0.25 (0.05)	(0.21− 0.31), 1.5	1.73 (0.60)	(1.03− 2.14), 2.1	1.63 (0.54)	(0.97− 2.14), 2.2
H7	1.36 (0.53)	(0.27− 2.18), 8.1	–	–	1.42 (0.54)	(0.24− 2.18), 9.1
H8	2.44 (0.15)	(2.34− 2.55), 1.1	1.45 (0.53)	(0.40− 2.45), 6.1	3.88 (0.58)	(0.42− 2.57), 6.1

**Table 12:** Statistical ESAK (mGy) values for all centers and comparison to other studies and DRLs.

	*This Study*	*Previous Studies; mean and (max‐min)*									*DRLs*	
*Exam*	*mean, max‐min, max/min*	*Montenegro (2012)*	*Iran (2013)*	*Serbia (2005)*	*India (2010)*	*Iran (2007)*	*Malaysia (1998)*	*Korea (2007)*	*HPA (UK) (2005)*	*HPA (UK) (2010)*	*EC (1999)*	*IAEA (1996)*
Skull AP	1.72, (0.22− 4.47), 20.3	2.80 (0.2− 8.4)	6.84 (2.05− 8.65)	4.0 (0.6− 12.0)	–	2.79	–	2.04 (0.90− 3.43)	1.41	1.8	–	–
Skull PA	2.25, (0.19− 25.8), 135.8	–	6.84 (2.05− 8.65)	–	5.40	2.79	4.78	–	1.41	1.8	5	5
Skull LAT	1.5, (0.39− 3.63), 9.3	2.10 (0.2− 7.4)	7.89 (2.81− 9.70)	2.7 (1.0− 5.8)	4.11	1.57	3.34	1.50 (0.73− 2.46)	1.01	1.1	3	3
Chest AP	1.12, (0.08− 0.76), 9.5	–	–	–	0.38	–	–	–	0.13	0.16	–	–
Chest PA	1.0, (0.02− 5.06), 253.0	0.75 (0.05− 4.0)	0.74 (0.32− 1.95)	0.6 (0.1− 2.0)	0.53	0.37	0.28	0.21 (0.04− 0.58)	0.11	0.12	0.3	0.4
Chest LAT	2.20, (0.04− 13.74), 343.5	1.95 (0.06− 4.9)	2.21 (0.44− 3.90)	1.4 (0.3− 4.0)	1.58		1.4	1.56 (0.28− 5.72)	0.44	0.48	1.5	1.5

## IV. DISCUSSION

Widespread, fast‐growing demand for X‐ray examinations by physicians in the developing countries like Iran and the increasing number of X‐ray machines in the past 10 years have led to an increase in the number of X‐ray examinations per unit of population. In Iran, as a developing country, there is no established national diagnostic reference level in radiology centers. Also, Iran was not involved in the international patient dose survey project, under the supervision of the IAEA, in the year 2004, which makes these types of studies more important. Thus, a comprehensive national plan seems to be necessary, to be designed by national authorities, to determine NDRLs in X‐ray examinations performed in radiology centers in Iran.

Since there were no digital radiography systems, the study pertained to only conventional radiography devices and different X‐ray units were included in the survey. This survey reveals significant variations in radiological practice. Exposure parameters are set by radiologic technologists, which change in a wide range; e.g., the tube loading (mAs) varied from 2 to 80 for skull PA examinations, and from 2 to 202 for chest LAT examinations. Substantial variations are recorded in FFD for the same procedures. For example, FFD has changed from 50 to 180 cm for skull LAT projection, and other examinations also show similar significant variations. These considerable variations in exposure parameters have led to great differences in mean values of ESAK for the same procedures; up to a factor of 20 across all hospitals (chest LAT), and of 28 within hospitals (chest LAT in H5 center). The max to min ratio in chest PA and LAT procedures among all hospitals showed extremely large differences — 253 and 344, respectively.

IAEA TECDOC 1423 indicates that beam filtration, tube potential, SF speed class, mAs, and film processing are the interrelated factors affecting patient dose. Patient dose could not be attributed to these parameters alone, but even focal‐film distance, patient size, tube output, exposure parameters setting mode, daily workload, equipment age, and developer and fixer solution replacing date could be partly related.

The minimum HVL in a radiographic X‐ray tube for use up to 70 kVp should not be less than 2.1 mm of Al eq. Filtration more than the minimum value can reduce ESAK, but significant increase in the HVL due to more additional filters would adversely affect image contrast through beam hardening. It also leads to high patient dose and the tube overheating by greater mAs applied to compensate radiation intensity on the table top. All radiology systems in this study met the minimum HVL requirement, except the H3 center.

All hospitals used tube voltages lower than European Guidelines.^(20)^ Use of the high kV technique reduces patient dose, but is not desirable in cases where high contrast image is needed. Based on this guideline, recommended kVp for both the chest PA and LAT examinations should be 125, but recorded values in this study were 67 and 71, respectively. There are several reasons why high applied voltages are not practical. The main reasons are device aging, as well as frequent repairs and replacement of the X‐ray tubes. Also, service provider companies, in the case of technical problems that are related to high‐voltage burden to the radiographic system, typically higher than 85 or 90 kilovoltages, do not provide any services. In other words, the radiology centers are forbidden to use kVps more than 85 to avoid damaging the tubes.

That is a limiting factor in providing imaging services to obese patients in the cases of higher kV procedures like lateral lumbar spine examinations. This problem can be solved by replacing older devices with new ones. Another important reason for low applied voltages is a wrong mindset of radiologist technologists about scattered and leakage radiation levels in a radiography control room, especially in the cases of high kVp X‐ray examinations. All centers also used lower FFDs in the chest projections than European standard.^(19)^ High mAs, as well as low applied voltages and FFDs, are the main reasons for the higher‐than standard ESAKs obtained, particularly in the chest projections. However, differences in patient size should be considered.

All X‐ray departments used 400‐speed SF cassettes. The speed class should be selected by the type of examination and 400‐speed class in chest imaging is justified. Use of the 400‐speed SF cassette combination contributes to reducing the ESAK in the skull projections in this study. The average age of the X‐ray machines used in this study was 15 years.

All hospitals in the study used manual exposure setting. Some centers had the AEC system, but it had been deliberately disconnected. This is in part due to lack of trained staff in the use of the AEC system. Manual exposure setting is an experience‐related skill and inexperienced staffs use a radiographic technique chart that was provided and posted near the control panel. In hospitals with a manual exposure setting, various exposure parameters were observed, which can be the reason for great differences in ESAK in the same procedures.

As Table 3 shows, the tube outputs were measured at 5 kVp intervals from 55 kV to 80 kV at 10 mAs. As Table 3 shows, the H6 and H3 X‐ray units delivered poor outputs at 80 kVp; 20.2 and 104.3 μGy/mAs, respectively. The reason for such a big difference (more than five times) in the tube outputs is insufficient filtration in the H3 unit despite being recently installed; and using a single phase generator in the H6 unit, as well as its age (24 years). In this study the age of all radiography equipment was more than 10 years, except the new H3 unit. Five units were older than the average (15 years). It is worth emphasizing that the age of the radiography equipment is not always the decisive factor for patient doses. However, in very old units, a small fraction of radiation leakage emanating from the X‐ray tube housing is less likely to expose radiology staff.

There was no clear association between daily workload and patient doses. All radiographs taken in this study were processed by automatic wet‐film processor systems in darkroom. Computed radiography (CR) systems were not included in the survey. Also, developer and fixer solution replacing dates may affect exposure parameters. The fresher the processing solutions the slightly lower the exposure parameters, so the time interval between the solution replacing date and the date in which radiography is performed should be considered.

As Table 12 shows, the mAs values obtained in this study for all chest projections are higher than those of obtained in the UK, Malaysia, Korea, and India, and even than in previous studies performed in Iran (2003 and 2007). Also, the tube kVps reported in our study for all chest projections are greater than the corresponding values in India and Iran (2007) and lower than Malaysia and two the UK studies (2005 and 2010). The tube kilovoltages obtained in this study for skull projections are lower than India, Malaysia, Korea, and two the UK studies and comparable to Iran (2007). As Table 12 shows, the results of this study revealed that the mean ESAKs of patients in Khuzestan province in three skull projections were lower than the IAEA and EC dose reference levels, 1.5, 1.72, and 2.25 mGy for skull LAT, skull AP, and skull PA, respectively, as well as the following studies: Iran (2003 and 2009), Montenegro, India, Serbia, Malaysia, and Korea with the exception of the UK (2005 and 2010) mean values. The study also showed that patient doses in all chest projections exceed the EC and IAEA DRLs — 1.0, 1.12, and 2.20 mGy for chest PA, chest AP, and chest LAT, respectively — and ESAKs of the previous studies with the exception of chest LAT that is comparable to Iran (2013) findings. The mean ESAK in chest PA projection were 2.5, 3, 8, and 9 times greater than the IAEA, EC, UK (2000), and UK (2005) DRLs, respectively. Likewise, the chest LAT projection showed in a similar manner, but it was slightly lower. Comparison of ESAK values of skull AP and chest AP with IAEA and EC dose reference levels are not possible as there are no available DRLs.

## V. CONCLUSIONS

This survey should be extended to include digital radiography systems, CT scans, and interventional radiology procedures to establish a local dose reference levels and there should be periodical review of the values of the LDRLs to ensure that they remain appropriate. The authors believe that this study provides evidence that dose reduction in the simple X‐ray examinations is feasible. Special consideration should be given to adequate training of imaging staffs, to updating clinical audits and patient dose considerations, to implementation of systematic and regular QA and QC programs, and to the use of qualified diagnostic medical physicists in medical imaging departments to optimize radiological practice. Therefore, the authors recommend that DRLs obtained in this study can be used as local DRL.

## ACKNOWLEDGMENTS

This project was funded by Behbahan Faculty of Medical Sciences based on research project No. 9302.
